# Computational Identification of Uncharacterized Cruzain Binding Sites

**DOI:** 10.1371/journal.pntd.0000676

**Published:** 2010-05-11

**Authors:** Jacob D. Durrant, Henrik Keränen, Benjamin A. Wilson, J. Andrew McCammon

**Affiliations:** 1 Biomedical Sciences Program, University of California San Diego, La Jolla, California, United States of America; 2 Department of Cell and Molecular Biology, Biomedical Center, Uppsala University, Uppsala, Sweden; 3 University of California San Diego, La Jolla, California, United States of America; 4 Department of Ecology & Evolutionary Biology, University of Arizona, Tucson, Arizona, United States of America; 5 Department of Chemistry & Biochemistry, National Science Foundation Center for Theoretical Biological Physics, National Biomedical Computation Resource, University of California San Diego, La Jolla, California, United States of America; 6 Department of Pharmacology, University of California San Diego, La Jolla, California, United States of America; 7 Howard Hughes Medical Institute, University of California San Diego, La Jolla, California, United States of America; McGill University, Canada

## Abstract

Chagas disease, caused by the unicellular parasite *Trypanosoma cruzi*, claims 50,000 lives annually and is the leading cause of infectious myocarditis in the world. As current antichagastic therapies like nifurtimox and benznidazole are highly toxic, ineffective at parasite eradication, and subject to increasing resistance, novel therapeutics are urgently needed. Cruzain, the major cysteine protease of *Trypanosoma cruzi*, is one attractive drug target. In the current work, molecular dynamics simulations and a sequence alignment of a non-redundant, unbiased set of peptidase C1 family members are used to identify uncharacterized cruzain binding sites. The two sites identified may serve as targets for future pharmacological intervention.

## Author Summary

Chagas disease, an infection that afflicts millions of people in Central and South America, is caused by the unicellular parasite *Trypanosoma cruzi*. In the chronic stage of the disease, patients' hearts are adversely affected. Chagas is the leading cause of infectious heart disease in the world. The current drugs used to treat Chagas disease are highly toxic, unable to eradiate the parasite, and subject to increasing drug resistance. Consequently, researchers are actively looking for new treatments. One attractive drug target is a Chagas protein called cruzain, which is required for the parasite's survival. Drugs that can inhibit the correct functioning of cruzain within the parasite may one day serve as powerful treatments in the fight against this devastating tropical disease. To design drugs that will be effective against cruzain, we need to know what portions of the protein are crucial for its functionality. For example, portions of the protein that bind to other proteins or to small molecules are likely to be critical. These regions are called “binding sites.” In the current work, we identify two uncharacterized cruzain binding sites. With this knowledge in hand, future researchers may be able to design drugs that target these sites.

## Introduction

American trypanosomiasis, also known as Chagas disease, is endemic to Central and South America, where 90 to 100 million people are at risk of infection [1], 10 to 20 million people are infected [1], [2], and 50,000 die annually [3]. The disease is caused by the unicellular parasite *Trypanosoma cruzi* (*T. cruzi*), an organism transmitted by insects of the *Reduviidae* family. After drawing a blood meal from its human host, the insect reflexively releases feces containing the parasite into the resulting wound [4]. Once blood borne, the parasites infiltrate host cells and replicate. Following replication and maturation, host cells burst open, releasing new *T. cruzi* parasites into the bloodstream [5].

The acute phase of the disease, which typically persists for two months and has a fatality rate of 2 to 8%, is characterized by the mononuclear inflammation and necrosis of parasitized cells, especially in the heart [6]. The chronic stage of the disease is characterized by dilated cardiomyopathy; indeed, American trypanosomiasis is the leading cause of infectious myocarditis in the world [7].

New therapies for Chagas disease are urgently needed. Current treatments, nitrofurans like nifurtimox and benznidazole, are highly toxic [6], [8], [9], and drug resistance has been reported [10]. Furthermore, one recent study demonstrated that these compounds neither eradicate the parasite nor prevent cardiomyopathy over the long term [11].

The major cysteine protease of *T. cruzi*, called cruzain or, alternatively, cruzipain, is one attractive drug target [12]. A member of the peptidase C1 protein family, cruzain is present and essential in all stages of *T. cruzi* development [2], [13]. Over-expression of cruzain enhances the transformation of the parasite into the infective form [14], and reduced protease activity prevents infection in wild-type mice [9]. Additionally, cysteine protease inhibitors block both the replication and the differentiation of the parasite *in vitro* and *in vivo* [12], [15]–[22]. Cruzain inhibitors can cure infection in cell, mouse, and dog models [18],[23].

The future rational design of improved cruzain inhibitors necessitates a better understanding of the flexibility and conformational changes characteristic of the cruzain active site. Molecular dynamics (MD) simulations, in which the forces that act on the atoms of a molecular system are approximated using Newton's laws of motion, can be powerful tools for better understanding protein flexibility and conformational sampling relevant to drug design. For example, one recent MD study of HIV integrase revealed a previously uncharacterized binding trench that was subsequently exploited in the design of Isentress (raltegravir), an HIV drug approved by the FDA in 2007 [24]. Importantly, this trench was not evident in the then available crystal structures; it was only by studying active-site flexibility *via* MD that the trench was initially identified.

Additional novel sites of enzymatic, allosteric, or structural importance can be identified computationally by comparing the sequence of the target protein with evolutionarily related enzymes. Critical protein residues are often conserved across multiple members of the same protein family; once multiple sequences are aligned, conserved patches of protein residues can be easily identified. Additional experimental studies can then characterize the pharmacological significance of these patches.

Given the urgent need for novel antichagastic therapeutics, we here use computational methods, including molecular dynamics (MD) simulations and a sequence alignment of a non-redundant, unbiased set of peptidase C1 family members, to identify previously uncharacterized binding regions that may serve as sites for future pharmacological intervention.

## Methods

### MD Simulations

To prepare cruzain for MD simulations, hydrogen atoms were added to a high-resolution cruzain crystal structure (PDB: 1ME4) [25] using PDB2PQR to approximate protein protonation at pH 5.5, the pH of the reservosome where cruzain is located in the epimastigote stage of the parasite [26]–[28]. Protonation states were subsequently verified manually. Hydrogen atoms were added to the bound hydroxymethyl-ketone inhibitor using Discovery Studio (Accelrys). The LEaP module of the AMBER9 suite [29] was used to solvate the system by submerging the protein in a TIP3P water box [30] that extended 10 Å beyond the protein in all directions. All crystallographic water molecules were maintained. Ten sodium cations were added to make the system electrically neutral; additional ions were then added to simulate a more physiological 20 mM NaCl solution. The system was parameterized using the generalized and FF99SB AMBER force fields [31], [32].

NAMD2.7b1 [33] was used for all MD simulations. Periodic boundary conditions were employed with the particle mesh Ewald method to account for electrostatic effects (smoothing cutoff: 14 Å). Langevin dynamics were applied to maintain the temperature, and a modified Langevin piston Nosé-Hoover thermostat was used to maintain 1 atm pressure. The initial structure was minimized in four distinct steps; hydrogen atoms were first relaxed for 5,000 steps; hydrogen atoms, water molecules, and ions were next relaxed for 5,000 steps; hydrogen atoms, water molecules, ions, and protein side chains were then relaxed for 10,000 steps; and, finally, all atoms were relaxed for 25,000 steps. Following minimization, the system was equilibrated with an NPT-ensemble at 310 K using stepwise harmonic-constraint force constants of 4, 3, 2, and 1 kcal/mol/Å^2^ on the protein backbone. 250,000 steps of MD simulation were executed for each force constant (1 fs time step).

Following minimization and equilibration, five distinct 20-ns productive runs were performed (10^7^ steps of 2 fs) with distinct random seeds in order to sample many protein configurations.

### Trajectory Clustering

The RMSD-based *gromos* clustering algorithm, as implemented in the GROMACS++ computer package (*g_cluster*), was used to cluster the conformations sampled during the five 20-ns MD simulations [34]. Structures were first extracted from the trajectories every 50 fs, generating 4,002 snapshots total. These snapshots were aligned by their C_α_ atoms and clustered on the 73 residues of the cruzain active site, defined as all residues within 10 Å of the ligand: 18–31, 50, 53–54, 57–72, 74, 91, 93–98, 115, 117, 120, 136–142, 144–145, 158–165, 181–184, 203–210.

The *gromos* clustering algorithm was first described by Daura et. al. [35]. In brief, for each protein conformation in a pool of conformations, the RMSD distance between the atoms of the aforementioned residues and the corresponding atoms of every other protein conformation in the pool (potential “neighbors”) is calculated. The conformation with the most neighbors within a user-specified distance cutoff (“close neighbors”) is then selected. This conformation, together with its close neighbors, constitutes the first cluster. The protein conformations of the first cluster are then removed from the pool, and the process is repeated with the remaining conformations until none are left.

When a cutoff of 0.95 Å was used, this procedure produced 24 clusters. The central member of each cluster was considered most representative; the set of all central members is said to constitute an *ensemble*.

### Calculating Beta Factors from the MD Simulation

To derive beta factors from the motions sampled during the MD simulations, all trajectories were concatenated, and the RMSF of each protein residue was calculated using the AMBER 9 *ptraj* module [29]. These RMSF values were converted into beta factors by multiplication, where β = RMSF * 8π^2^/3.

### Virtual Screening

A small-molecule library was prepared from the ligands of the NCI Diversity Set II, a set of freely available, diverse, drug-like molecules. The Schrödinger LigPrep program (Schrödinger) was used to assign protonation states at pH 5.5 and to identify and generate tautomers and stereoisomers. One ligand could not be processed with LigPrep; instead, hydrogen atoms were added to this ligand and its geometry was optimized using Discovery Studio (Accelrys).

The ligands of this small-molecule library were docked into a 1.20 Å cruzain crystal structure (PDB ID: 1ME4; [25]). Hydrogen atoms were added using PDB2PQR [27], [28] at pH 5.5. At this pH, C25 and H159 formed the thiolate/imidazolium pair required for the catalytic mechanism [36]. An initial virtual screen was performed using the CDOCKER docking software (Accelrys) with a docking sphere 15 Å in diameter centered on the coordinates of the crystallographic ligand, as that program was able to recapture the crystallographic poses of two known hydroxymethyl-ketone cruzain inhibitors [25]. The CDOCKER-predicted pose of each of the ligands was rescored using the PLP2 scoring function [37]. The best ligands as evaluated by PLP2 were compiled into a new small-molecule ligand library enriched for potential cruzain inhibitors.

To account for receptor flexibility, we subsequently used the relaxed-complex scheme [38], a protocol that has been used previously to identify inhibitors of FKBP [39], HIV integrase [24], and *T. brucei* RNA editing ligase 1 [40]. The compounds of the enriched small-molecule library were docked into the 24 members of ensemble, again using CDOCKER (Accelrys). Each of these compounds was rescored with the PLP2 [37] scoring function. For each ligand, a PLP2-based ensemble-average score was calculated according to the following equation:
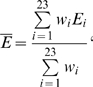
(1)where 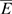 is the weighted ensemble-average score, *w_i_* is the size of cluster *i*, and *E_i_* is the best score of the ligand, independent of tautomeric or stereoisomeric form, docked into the centroid of cluster *i*.

### Alignment of a Non-Redundant, Unbiased Set of Peptidase C1 Family Members

Cruzain was compared to other members of the peptidase C1 family. First, the UniProt database [41] was used to identify reviewed members of the peptidase C1 family, as defined by the MEROPS classification [42], that had structures deposited in the Protein Data Bank [43]. All amenable sequences except those of cruzain were then aligned using ClustalW in the MultiSeq extension of VMD [44]–[46]. A non-redundant set was selected from these aligned peptidase C1 sequences (sequence QR: 75; GF: 1.0). Gaps in the sequences were then removed, and ClustalW was used to align the corresponding sequences to a cruzain crystal structure (PDB: 1AIM) that was chosen as a non-redundant structure from the set of all cruzain structures aligned using STAMP [47]. The following sequences were aligned: 1A6R, 1AEC, 1CJL, 1DEU, 1FWO, 1JQP, 1K3B, 1M6D, 1PCI, 1XKG, 2C0Y, 2CB5, 2FO5, 2O6X, 2WBF, 3PBH, 7PCK, and 8PCH. Residues were colored by similarity according to the BLOSSUM30 matrix.

## Results and Discussion

Motivated by the urgent need for novel antichagastic therapeutics, we set out to identify previously uncharacterized cruzain sites that might serve as future targets for pharmacological intervention. Five 20-ns MD simulations were first used to probe the dynamics of the cruzain active site, as knowledge of protein dynamics can provide important structural insights beyond the information that can be obtained from crystal structures alone.

### System Equilibration

**Figure 1:**
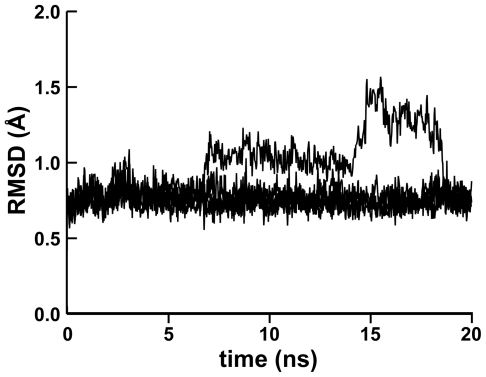
MD equilibration. The five MD trajectories were aligned by protein alpha-carbon atoms, and the RMSD of each trajectory relative to the first frame was calculated as a function of time. The protein N-terminal tail of the first simulation assumed several rotameric states. The remaining four simulations were equilibrated, as demonstrated by their convergent RMSD values.

While four of the five 20-ns MD simulations equilibrated, as judged by convergent RMSD values, the RMSD plot of the first simulation suggested that several conformational states had been sampled (Figure 1). A careful examination of the trajectory revealed that a mobile N-terminal tail was entirely responsible for the non-convergent RMSD values of the first simulation. In the crystal structure (PDB: 1ME4) [25], as in four of the five MD simulations, the N-terminal tail is held against the protein *via* hydrogen bonds between the A3 backbone carbonyl and the D167 backbone amine, and between the P2 backbone carbonyl and the Y166 side-chain hydroxyl group. In the first MD simulation, however, the hydrogen bond between P2 and Y166 broke after 6.7 ns. After 14.3 ns, the bond between A3 and D167 broke, allowing the N-terminal tail to rotate such that new hydrogen bonds were formed between the D167 side-chain carboxylate group and the backbone amines of both A3 and A4. After 18.7 ns, the N-terminal tail returned to its original position. While these conformational changes are interesting, they occur far from the peptide binding site and so are probably not relevant to drug design. Importantly, when the first three residues of the protein are omitted from the RMSD calculation, the RMSD plot of the first MD simulation is convergent, similar to the RMSD plots of the other four simulations.

### The Cruzain Active Site

**Figure 2:**
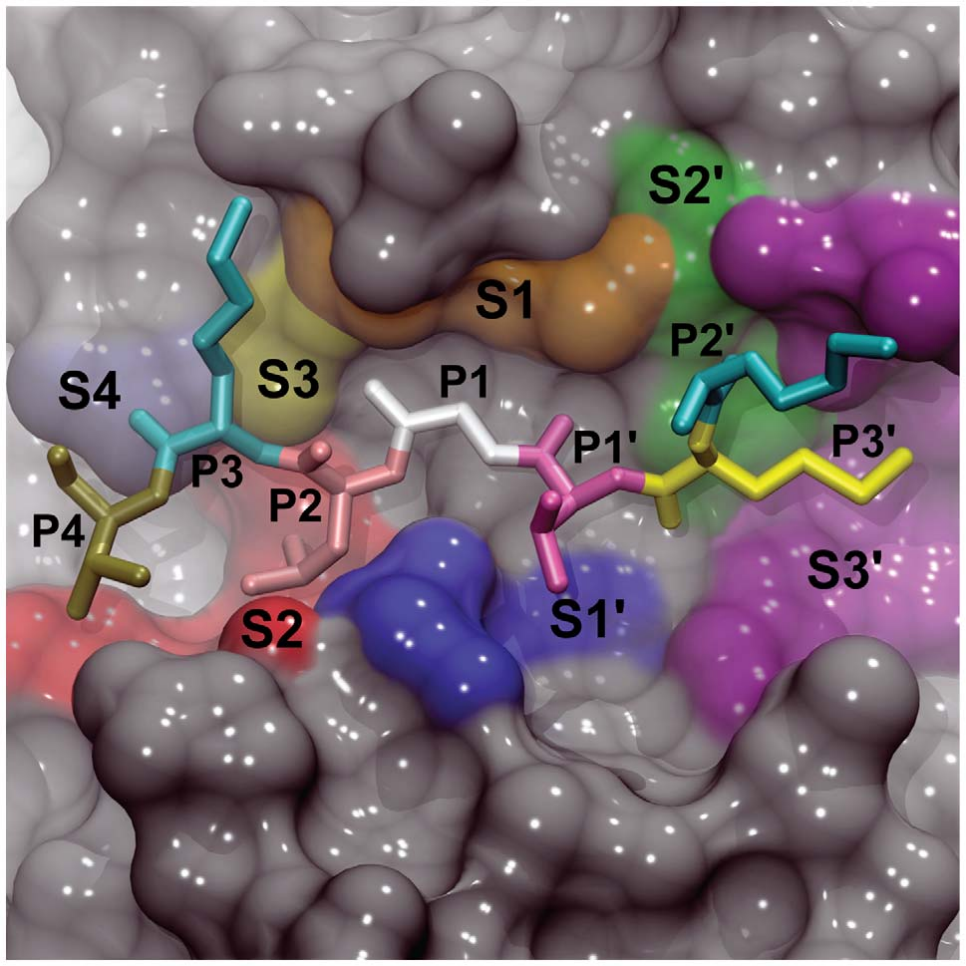
The cruzain active site colored according to the seven substrate-binding subsites. Four subsites on the acyl side of the cleaved peptide bond, named S4, S3, S2, and S1, bind the peptide amino acids P4, P3, P2, and P1. Three subsites on the amino side of the bond, named S1′, S2′, S3′, bind the peptide amino acids P1′, P2′, and P3′. As no crystal structure of cruzain bound to a peptide substrate was available, the peptide shown was taken from a crystal structure of the homologous protein procathepsin K (PDB: 1BY8).

The MD simulations were subsequently used to study the flexibility of the cruzain active site. Cruzain, like other cysteine proteases, contains seven subsites that bind peptide amino acids. Four subsites on the acyl side of the cleaved peptide bond, named S4, S3, S2, and S1, bind the peptide amino acids P4, P3, P2, and P1. Three subsites on the amino side of the bond, named S1′, S2′, S3′, bind the peptide amino acids P1′, P2′, and P3′ (Figure 2) [48]. The only well defined subsites of these seven are S2, S1, and S1′, and only S2 and S1′ demonstrate significant specificity [49].

To judge the flexibility of the cruzain active site, the beta factor of each protein residue was calculated from the molecular motions sampled during the MD simulation. In general, the active site was remarkable for its great stability, likely in part due to the bound hydroxymethyl-ketone inhibitor [25].

### Trajectory Clustering

To better distinguish between the many conformational states sampled by the MD simulations, 4,002 protein configurations were extracted from the simulations at regularly spaced intervals and grouped into 24 clusters by RMSD using the *gromos* clustering algorithm [35]. The centroid member was selected from each cluster, and the set of all centroid members, representative of the many conformations sampled by the MD simulations, is said to constitute an *ensemble*.

### Virtual Screening

**Figure 3:**
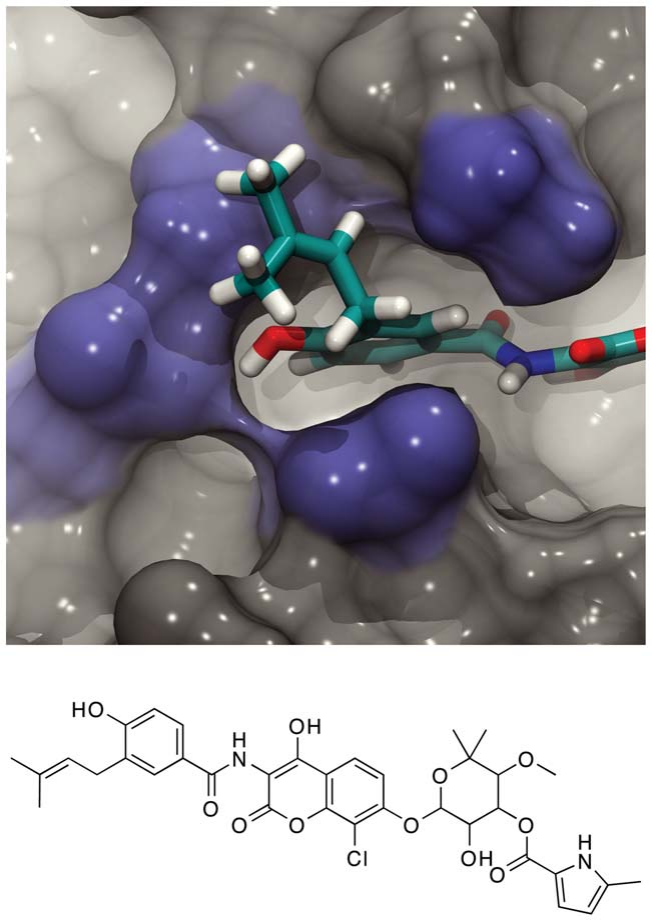
The binding of compound 1 to the semi-open conformation of cruzain. Compound **1** (clorobiocin, bottom), a known antagonist of *T. cruzi* amastigote growth, is predicted to occupy a previously uncharacterized binding pocket adjacent to the S2 subsite (top).

To test the potential physiological relevance of the ensemble-member active-site conformations, CDOCKER (Accelrys) was used to dock the compounds of the NCI Diversity set II, a set of freely available, diverse, drug-like molecules, into both the cruzain crystal structure and the 24 protein conformations of the ensemble. A full account of the results of this virtual screen is forthcoming; however, one of the predicted inhibitors warrants further discussion here. Compound **1** (clorobiocin, Figure 3) was the best predicted novel cruzain inhibitor as evaluated by the PLP2 scoring function [37] in both the screen against the static crystal structure and the relaxed-complex screen against the ensemble of 24 conformations. As positive controls, two hydroxymethyl-ketone inhibitors (PDB: 1ME3) [25] were included in the relaxed-complex screen. After rescoring with an ensemble-average PLP2 score, these compounds ranked even better than compound **1**, confirming that the PLP2 scoring function is well suited to this particular protein receptor.

We note with interest that previous studies have demonstrated that compound **1** antagonizes *T. cruzi* amastigote growth [50], [51]. The primary protein target of clorobiocin is thought to be *T. brucei* topoisomerase II, but the idea of a polypharmacophoric mechanism that includes cruzain inhibition is interesting. The PLP2 scores of compound **1** docked into the central members of the first, second, and third most populated clusters were 95.07, 117.7, and 115.68, respectively.

To understand why compound **1** binding to the second ensemble conformation was favored, the pose of the ligand docked into that conformation was analyzed. While docking poses should never be blindly accepted, this particular pose seemed promising. Aside from having the best PLP2 score, this binding mode placed a conjugated ring in the S2 pocket, similar to the binding modes of some known ligands (e.g. some vinyl sulfone inhibitors [52]) and of some native substrates [53]. Importantly, the docked pose also suggested that one of the ligand rings binds in a previously uncharacterized, druggable pocket immediately beyond the S2 subsite (Figure 3).

### An Additional Binding Pocket Beyond the S2 Subsite

**Figure 4:**
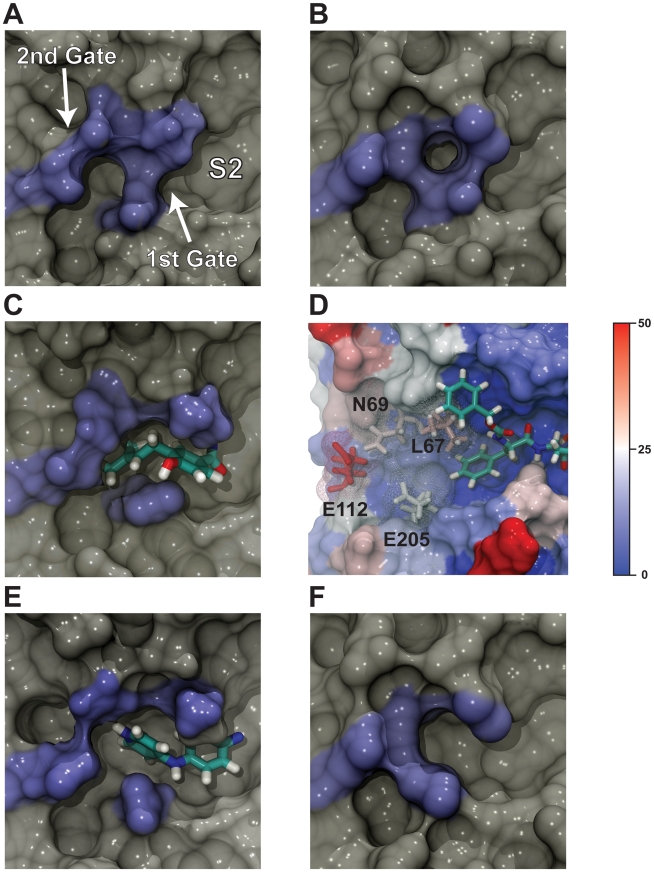
A previously uncharacterized binding pocket adjacent to the S2 subsite. The protein conformations depicted in A, B, C, E, and F are shown from the same orientation. Molecular fragments were excised from selected members of the NCI diversity set docked into semi-open and fully open protein conformations extracted from the MD simulation. A) The central member of the first cluster extracted from the MD simulation, in which the pocket is closed. The S2 subsite, as well as the first and second gate, are labeled. B) A cruzain crystal structure (PDB: 2EFM) in which the pocket is likewise closed. C) The central member of the second cluster, in which the pocket is semi-open. A small molecular fragment is shown docked into the pocket to demonstrate druggability. D) The cruzain active site with residues colored according to beta values calculated from the MD simulation. Blue indicates stability, and red indicates mobility. Several of the residues that comprise the previously uncharacterized pocket are flexible. E) The central member of the third cluster, in which the pocket is fully open. A small molecular fragment is shown docked into the pocket to demonstrate druggability. F) A cruzain crystal structure (PDB: 1ME3) in which the pocket is semi-open.

The beta factors of the protein residues that from this previously uncharacterized pocket revealed significant protein flexibility. Two of the residues that form the distal wall of the S2 subsite, L67 and E205, were somewhat flexible (Figure 4D), and two other protein residues beyond the S2 subsite, N69 and E112, were also mobile (Figure 4D). Together, these four flexible residues comprise two “gates” (L67-E205 and N69-E112) that, when open, form the walls of a previously uncharacterized druggable pocket that medicinal chemists have yet to exploit.

Published cruzain crystal structures hint at the existence of this additional pocket. A crystal structure of cruzain bound to a vinyl sulfone derived inhibitor (PDB: 2EFM) demonstrates a closed configuration (Figure 4B), while a crystal structure of cruzain bound to a hydroxymethyl-ketone inhibitor (PDB: 1ME3) [25] demonstrates a semi-open configuration (Figure 4F).

The crystal structures, however, do not fully capture the extent of opening demonstrated by the MD simulations. The central member of the top cluster, which accounted for 82.5% of the trajectory, had a closed conformation (Figure 4A). The central member of the second cluster, accounting for 6.9% of the trajectory, had a semi-open conformation (Figure 4C), and the central member of the third cluster, accounting for 3.6% of the trajectory, was fully open (Figure 4E). As shown in Figures 4C and 4E, molecular docking demonstrates that both the semi-open and the fully open conformations can easily accommodate small molecular fragments.

### The First Gate

**Figure 5:**
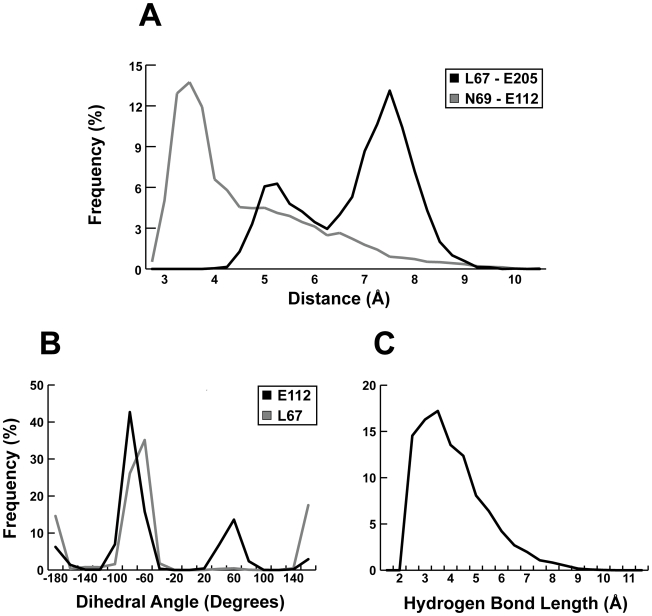
The opening and closing of the two “gates” that comprise the previously uncharacterized pocket. A) Histograms of the distances between the L67 γ carbon atom and the E205 δ carbon atom (the first gate, in black), and the distances between the N69 side-chain amino nitrogen atom and the E112 δ carbon atom (the second gate, in gray), over all 100 ns of trajectory. When the L67-E205 distance is ∼7.5 Å, the first gate is open. When ∼5.5 Å, the gate is closed. Similarly, when the N69-E112 distance is ∼6.0 Å, the second gate is open; when ∼3.5 Å, the gate is closed. B) Histograms of the dihedral angles defined by the backbone amino nitrogen atom and the α, β, and γ carbon atoms of L67 (in gray) and E112 (in black), respectively, measured over the course of the trajectory. The first gate is open when the L67 dihedral angle is ∼60° and closed when ∼180°. The second gate is open when the E112 dihedral angle is 60° or 180°, and closed when −60°. C) A histogram of the distances between the N69 side-chain amino nitrogen atom and the E112 carboxylate oxygen atoms, demonstrating the formation of a transient hydrogen bond.

To further characterize the opening and closing of the first gate, the distance between the L67 γ carbon atom and the E205 δ carbon atom (*d_1_*) was monitored over all 100 ns of trajectory. A histogram of these distances was bimodal (Figure 5A) and suggested that the gate was open (*d_1_*>6.25 Å) 70% of the time (*d_1_* = 7.6 Å±0.6) and closed (*d_1_*<6.25 Å) 30% of the time (*d_1_* = 5.4 Å±0.4). As a reference, this same distance is 7.8 Å and 8.4 Å in the semi-open and fully open conformations, respectively, both of which can accommodate a ligand (Figures 4C and 4E).

We note that the presence of the hydroxymethyl-ketone inhibitor may have affected the dynamics of the first gate by largely immobilizing the E205 residue in an open conformation. E205 plays a unique role in substrate binding. The cruzain S2 subsite, like that of cathepsin B, differs from other cysteine proteases in that it can bind both hydrophobic and basic amino acids [2], [53]. The E205 residue acts as a highly mobile switch. When a basic amino acid like arginine occupies the S2 subsite, the acidic E205 carboxylate group swings into S2 to interact with the guanidino group, a conformation that can be seen in the crystal structure of cruzain bound to benzoyl-arginine-alanine-methyl ketone (PDB: 2AIM) [2], [53]. When a hydrophobic amino acid occupies the S2 subsite, E205 rotates away from S2 and interacts with the solvent [2], [53], a conformation evident in the crystal structure of cruzain bound to WRR-99 (PDB: 1EWL).

The hydrophobic phenyl group of the hydroxymethyl-ketone inhibitor present in the S2 subsite of the MD simulation locked E205 in the open, solvent exposed conformation (Figure 4D). Consequently, the dynamics of the first gate were mostly determined by L67. A histogram of the dihedral angle defined by the backbone amino nitrogen atom and the α, β, and γ carbon atoms of L67, measured over the course of the trajectory, demonstrated that the side chain of this important residue rotated freely (Figure 5B). Visual inspection confirmed that gate opening occurred when the dihedral angle (θ_1_) was roughly −60° (*d_1_* = 7.6 Å±0.7 when −140°<θ_1_<60°), and that gate closing occurred when the dihedral angle was roughly 180° (*d_1_* = 5.7 Å±0.7 when θ_1_<−140° or θ_1_>60°). By this metric, the first gate was open 67% of the time, a value that matches that found by measuring the distance between the L67 γ carbon atom and the E205 δ carbon atom directly.

### The Second Gate

To assess the opening and closing of the second gate, the distance between the N69 side-chain amino nitrogen atom and the E112 δ carbon atom (*d_2_*) was monitored over all 100 ns of trajectory. A histogram of these distances was again bimodal (Figure 5A) and suggested that the second gate is open (*d_2_*>4.5 Å) 43% of the time (*d_2_* = 6.0 Å±1.1), and closed (*d_2_*<4.5 Å) 57% of the time (*d_2_* = 3.7 Å±0.4). As a reference, this same distance is 5.3 Å in the fully open conformation, which can accommodate a ligand (Figure 4E).

Both N69 and E112, which form the second gate, are mobile. Of these two residues, E112 is particularly flexible (Figure 4D). Visual inspection of the trajectory confirmed that N69 and E112 interact with each other *via* a transient hydrogen bond between the N69 side-chain amino nitrogen atom and the E112 carboxylate oxygen atoms (Figure 5C). A hydrogen bond between these two residues (distance cutoff of 3.5 Å) was present in roughly 30% of the frames extracted from the trajectory.

As this hydrogen bond was transient, E112 often flipped out into the solvent, where the carboxylate group interacted with water molecules. A histogram of the dihedral angle defined by the backbone amino nitrogen atom and the α, β, and γ carbon atoms of E112 (θ_2_), measured over the course of the trajectory, confirmed that the side chain of this important residue can freely rotate (Figure 5B). Visual inspection demonstrated that gate opening occurred at two rotameric states, when the dihedral angle was 60° or 180° (*d*_2_ = 5.9 Å±1.4 when θ_2_>0° or θ_2_<−140°). Gate closing occurred when the dihedral angle was −60° (*d_2_* = 4.1 Å±0.9 when −140°<θ_2_<0°). By this metric, the second gate was open 34% of the time.

### Types of Fragments That Bind This Previously Uncharacterized Pocket

To determine what kinds of molecular fragments would best fit into the previously uncharacterized pocket immediately beyond the S2 subsite, we examined the predicted binding poses of NCI compounds docked into the third (fully open) ensemble conformation (Figure 4E). Roughly two-dozen ligands were predicted to occupy the previously uncharacterized pocket and to bind cruzain with high affinity. With some exceptions, the molecular fragments occupying the previously uncharacterized pocket were generally aromatic rings or aliphatic chains, often with hydroxyl groups that formed hydrogen bonds with the E205 carboxylate oxygen atoms. Numerous FDA-approved drugs contain hydroxylated rings (e.g. masoprocol, carbidopa, acetaminophen, etc.) and/or aliphatic chains (e.g. penciclovir, ethambutol, and miglitol), and so these fragments can be considered drug like.

### Alignment of a Non-Redundant, Unbiased Set of Peptidase C1 Family Members

Having used MD simulations to identify a previously uncharacterized binding pocket immediately beyond the S2 subsite, we next used a bioinformatics approach to identify other possible sites of importance. Residues critical to protein function, like those of an enzymatic or allosteric active site, like those that participate in essential protein-protein interactions, or like those that play important structural roles, are often conserved across multiple homologous members of the same protein family. To identify these critical residues, cruzain was compared to other members of the peptidase C1 family.

**Figure 6:**
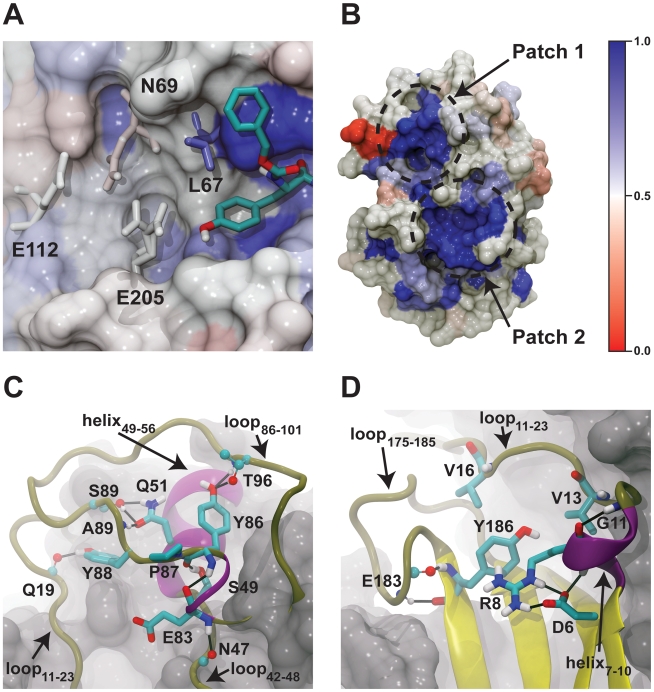
Cruzain (PDB: 1AIM) colored by residue conservation across multiple members of the peptidase C1 family. Conserved residues are shown in blue, and nonconserved residues are shown in red. A) The residues of the seven peptide-binding subsites are generally conserved. The S2 subsite, critical for specificity, is an important exception. Additionally, one of the residues of the previously uncharacterized binding pocket immediately beyond the S2 subsite, L67, is well conserved, while the remaining three residues of the pocket, N69, E112, and E205, are not. B) Two patches of highly conserved residues on the side of the protein opposite the proteolytic active site can be seen. The first, patch one, is comprised of Y88, P87, E83, Y86, Q51, and S49. The second, patch two, is comprised of Y186, R8, V16, G11, D6, and V13. C) A close-up view of the first patch. Conserved residues are shown in licorice, and non-conserved residues are shown in balls and sticks. Side chains or backbone atoms that do not participate in hydrogen bonds have been removed for the sake of clarity. D) A similar close-up view of the second conserved patch.

As expected, the residues of the seven subsites of the proteolytic binding pocket are generally conserved (Figure 6A, S1). The S2 subsite, critical for specificity, is an important exception; this site, like the S2 subsite of cathepsin B, differs from other cysteine proteases in that it can bind both hydrophobic and basic amino acids [2], [53].

Additionally, the six cysteine residues involved in disulfide bonds are conserved, suggesting that these bonds are critical for protein tertiary structure. A natural mutation in human cathepsin C, a related cysteine protease with the same papain fold, confirms this importance. Patients with a cathepsin C C291Y mutation, equivalent to a cruzain C56Y mutation, develop Papillon-Lefèvre syndrome due to cathepsin C dysfunction [54].

Surprisingly, there are two patches of highly conserved residues on the side of the protein opposite the proteolytic active site. The first, patch one, is comprised of Y88, P87, E83, Y86, Q51, and S49. The second, patch two, is comprised of Y186, R8, V16, G11, D6, and V13 (Figure 6B, S1). Both of these patches lie in a long shallow groove, formed largely by several disordered loops, which traverses the protein surface (Figure 6B). These loops include the loops spanning G11 to G23 (loop_11–23_), G42 to S48 (loop_42–48_), Y86 to T101 (loop_86–101_), and N175 to G185 (loop_175–185_). Though disordered, these loops are held rigid by the conserved residues of the two patches, which bind the loops to stable tertiary structures and/or to each other. This rigidity may serve to maintain the shape of the traversing groove.

To the best of our knowledge, this groove and its associated conserved patches, which are common to members of the peptidase C1 family, have not been previously characterized. These highly conserved patches may play roles in allosteric regulation or structural stability. Additionally, the shallow traversing groove formed by these two patches may also constitute a surface amenable to protein binding.

We first turned our attention to the first patch of conserved residues. S49 and Q51 are highly conserved buried residues that belong to a stable helix spanning S49 to L56 (helix_49–56_). Interactions between these residues and residues of loop_86–101_ help to pin the loop against the stable helix, thereby imparting stability to part of the traversing groove. The S49 side-chain hydroxyl group hydrogen bonds with the backbone carbonyl oxygen of Y86, another conserved residue. Additionally, the side-chain carbonyl oxygen atom of Q51 forms two hydrogen bonds, one with the backbone amine of A89 and one with the side-chain hydroxyl group of S89. The side-chain amine of Q51 hydrogen also hydrogen bonds with the side-chain hydroxyl group of S89 (Figure 6C).

Though cruzain mutagenesis data is absent from the literature, studies of the closely related human cathepsin C protein likewise suggest that S49 and Q51 have important roles. Patients with a cathepsin C S284N mutation, analogous to a cruzain S49N mutation, develop Papillon-Lefèvre syndrome [55], and patients with a cathepsin C Q286R mutation, analogous to a cruzain Q51R mutation, develop Haim-Munk syndrome. Both these syndromes are caused by cathepsin C dysfunction [56].

Y86 and P87, also conserved residues of the first patch, likewise seem to play an important role in imparting rigidity to the disordered loop_86–101_. The Y86 side-chain hydroxyl group forms two hydrogen bonds with T96, helping to maintain the hairpin shape of loop_86–101_. P87 does not participate in any hydrogen bonds, but the conformational rigidity of the proline backbone may contribute to the overall rigidity of loop_86–101_ as well (Figure 6C).

The rigidity of loop_86–101_ is in part transferred to loop_11–23_ and loop_42–48_ *via* the conserved residues Y88 and E83, respectively. The Y88 side-chain hydroxyl group hydrogen bonds with the Q19 backbone carbonyl oxygen atom, and the E83 backbone amine hydrogen bonds with the N47 backbone carbonyl oxygen atom, thereby holding all these loops rigid relative to one another. It is also interesting to note that the side-chain carboxylate group of N47 is solvent exposed and potentially capable of interacting with other proteins or small-molecule compounds that may bind in the traversing groove (Figure 6C).

We next turned our attention to the second patch of conserved residues. The conserved residues of this patch likewise serve to hold disordered loops rigid against underlying secondary structures. For example, a hydrogen bond exists between the backbones of two highly conserved residues, G11 and R8, that anchors part of loop_11–23_ to a small helix spanning W7 to R10 (helix_7–10_). Helix_7–10_ is in turn positioned relative to an underlying beta sheet by multiple hydrogen-bond interactions between the conserved residues R8 and D6. These interactions are likely critical for protein function; D6 is analogous to the cathepsin C residue D236, and patients with D236Y mutations develop Papillon-Lefèvre syndrome, again suggesting cathepsin C dysfunction (Figure 6D) [54].

The conserved residues of the second patch also impart some structure and rigidity to loop_175–185_. The backbone of the conserved residue Y186, part of a stable underlying beta sheet, forms two hydrogen bonds with the backbone of E183. These interactions not only hold part of loop_175–185_ fixed relative to the beta sheet, but also help stabilize a sharp turn at the sheet-loop junction. Additionally, the phenol group of Y186 forms an interesting π-cation interaction with R8, also conserved. This interaction may help impart curvature to the underlying beta sheet, contributing to the overall curvature of the traversing groove (Figure 6D).

Two additional conserved residues of the second patch, V13 and V16, do not participate in any hydrogen-bond interactions and have no obvious structural importance. Nevertheless, V16 likely has a critical, albeit unknown, role in protein function. V16 is analogous to the cathepsin C residue V249. Patients with V249F mutations develop Papillon-Lefèvre syndrome, again suggesting cathepsin C dysfunction [57]. Clearly, additional research is needed to further characterize these conserved patches and the traversing groove in which they are located (Figure 6D).

If we accept the hypothesis that *in vivo* the traversing groove constitutes a surface positioned at an important protein-protein interface, small molecules that target specific residues critical for protein binding may be able to disrupt the protein-protein interaction and potentially inhibit cruzain function. We note, however, that many of the residues that form the traversing groove are homologous with residues of human cathepsin C, and so relevant cruzain inhibitors are likely to inhibit cathepsin C as well. However, several residues, located between the two conserved patches (Figure 6B), are not themselves conserved. For example, the cathepsin C residues homologous to cruzain A15 and N47 are I258 and P224, respectively. It may therefore be possible to design cruzain-specific inhibitors that bind to non-conserved residues like A15 and N47.

### Conclusion

Chagas disease, caused by the unicellular parasite *T. cruzi*, claims 50,000 lives annually [3] and is the leading cause of infectious myocarditis in the world [7]. As current antichagastic therapies like nifurtimox and benznidazole are highly toxic [6], [8], [9], ineffective at parasite eradication [11], and subject to increasing resistance [10], novel therapeutics are urgently needed.

Cruzain, the major cysteine protease of *T. cruzi*, is one attractive drug target [12]. In order to further the development of cruzain inhibitors, we here used MD simulations to identify a previously uncharacterized druggable pocket adjacent to the S2 subsite and a sequence alignment of a non-redundant, unbiased set of peptidase C1 family members to identify two conserved patches that may play roles in allosteric regulation, structural stability, or protein-protein interactions.

Future directions include using computer-aided drug design to identify and characterize cruzain inhibitors that exploit the previously uncharacterized pocket immediately beyond the S2 subsite. Considerably more effort is required to characterize and exploit the two conserved patches opposite the peptide-binding site. While several of the residues of these patches are known to be critical for the function of cathepsin C, a cruzain homologue, mutagenesis studies are needed to directly confirm that they play an essential role in cruzain function as well. Once established as important, experiments are needed to further characterize the role these patches play. Two-hybrid screening or co-immunoprecipitation may identify other *T. cruzi* proteins that interact with cruzain. X-ray crystallography could then be used to determine whether or not these protein partners bind to the traversing groove formed by the two conserved patches identified in the current study. Additionally, small-molecule compounds that bind these patches may be useful tools for probing possible allosteric effects and/or disrupting critical protein-protein interactions.

## Supporting Information

Figure S1 The alignment of selected peptidase C1 family members.
(0.18 MB PDF)

## References

[1] WHO (2002). Control of Chagas disease.. World Health Organ Tech Rep Ser.

[2] Polticelli F, Zaini G, Bolli A, Antonini G, Gradoni L (2005). Probing the cruzain S2 recognition subsite: a kinetic and binding energy calculation study.. Biochemistry.

[3] Senior K (2007). Chagas disease: moving towards global elimination.. Lancet Infect Dis.

[4] McKerrow JH, Caffrey C, Kelly B, Loke P, Sajid M (2006). Proteases in parasitic diseases.. Annu Rev Pathol.

[5] Du X, Guo C, Hansell E, Doyle PS, Caffrey CR (2002). Synthesis and structure-activity relationship study of potent trypanocidal thio semicarbazone inhibitors of the trypanosomal cysteine protease cruzain.. J Med Chem.

[6] Rodriques Coura J, de Castro SL (2002). A critical review on Chagas disease chemotherapy.. Mem Inst Oswaldo Cruz.

[7] Bonney KM, Engman DM (2008). Chagas heart disease pathogenesis: one mechanism or many?. Curr Mol Med.

[8] de Castro SL (1993). The challenge of Chagas' disease chemotherapy: an update of drugs assayed against Trypanosoma cruzi.. Acta Trop.

[9] McKerrow JH, Doyle PS, Engel JC, Podust LM, Robertson SA (2009). Two approaches to discovering and developing new drugs for Chagas disease.. Mem Inst Oswaldo Cruz.

[10] Wilkinson SR, Taylor MC, Horn D, Kelly JM, Cheeseman I (2008). A mechanism for cross-resistance to nifurtimox and benznidazole in trypanosomes.. Proc Natl Acad Sci U S A.

[11] Lauria-Pires L, Braga MS, Vexenat AC, Nitz N, Simoes-Barbosa A (2000). Progressive chronic Chagas heart disease ten years after treatment with anti-Trypanosoma cruzi nitroderivatives.. Am J Trop Med Hyg.

[12] McKerrow JH, McGrath ME, Engel JC (1995). The cysteine protease of Trypanosoma cruzi as a model for antiparasite drug design.. Parasitol Today.

[13] Harth G, Andrews N, Mills AA, Engel JC, Smith R (1993). Peptide-fluoromethyl ketones arrest intracellular replication and intercellular transmission of Trypanosoma cruzi.. Mol Biochem Parasitol.

[14] Tomas AM, Miles MA, Kelly JM (1997). Overexpression of cruzipain, the major cysteine proteinase of Trypanosoma cruzi, is associated with enhanced metacyclogenesis.. Eur J Biochem.

[15] McKerrow JH, Engel JC, Caffrey CR (1999). Cysteine protease inhibitors as chemotherapy for parasitic infections.. Bioorg Med Chem.

[16] McKerrow JH (1999). Development of cysteine protease inhibitors as chemotherapy for parasitic diseases: insights on safety, target validation, and mechanism of action.. Int J Parasitol.

[17] Cazzulo JJ (2002). Proteinases of Trypanosoma cruzi: patential targets for the chemotherapy of Changas desease.. Curr Top Med Chem.

[18] Engel JC, Doyle PS, Hsieh I, McKerrow JH (1998). Cysteine protease inhibitors cure an experimental Trypanosoma cruzi infection.. J Exp Med.

[19] Engel JC, Doyle PS, Palmer J, Hsieh I, Bainton DF (1998). Cysteine protease inhibitors alter Golgi complex ultrastructure and function in Trypanosoma cruzi.. J Cell Sci.

[20] Chambers AF, Matrisian LM (1997). Changing views of the role of matrix metalloproteinases in metastasis.. Journal of the National Cancer Institute.

[21] Colasanti M, Salvati L, Venturini G, Ascenzi P, Gradoni L (2001). Cysteine protease as a target for nitric oxide in parasitic organisms.. Trends Parasitol.

[22] Ascenzi P, Salvati L, Bolognesi M, Colasanti M, Polticelli F (2001). Inhibition of cysteine protease activity by NO-donors.. Curr Protein Pept Sci.

[23] Barr SC, Warner KL, Kornreic BG, Piscitelli J, Wolfe A (2005). A cysteine protease inhibitor protects dogs from cardiac damage during infection by Trypanosoma cruzi.. Antimicrob Agents Chemother.

[24] Schames JR, Henchman RH, Siegel JS, Sotriffer CA, Ni H (2004). Discovery of a novel binding trench in HIV integrase.. Journal of medicinal chemistry.

[25] Huang L, Brinen LS, Ellman JA (2003). Crystal structures of reversible ketone-based inhibitors of the cysteine protease cruzain.. Bioorg Med Chem.

[26] Soares MJ (1999). The reservosome of Trypanosoma cruzi epimastigotes: an organelle of the endocytic pathway with a role on metacyclogenesis.. Mem Inst Oswaldo Cruz.

[27] Dolinsky TJ, Czodrowski P, Li H, Nielsen JE, Jensen JH (2007). PDB2PQR: expanding and upgrading automated preparation of biomolecular structures for molecular simulations.. Nucleic Acids Res.

[28] Dolinsky TJ, Nielsen JE, McCammon JA, Baker NA (2004). PDB2PQR: an automated pipeline for the setup of Poisson-Boltzmann electrostatics calculations.. Nucleic Acids Res.

[29] Case DA, Cheatham TE, Darden T, Gohlke H, Luo R (2005). The Amber biomolecular simulation programs.. J Comput Chem.

[30] Jorgensen WL, Chandrasekhar J, Madura JD, Impey RW, Klein ML (1983). Comparison of simple potential functions for simulating liquid water.. The Journal of chemical physics.

[31] Wang J, Wolf RM, Caldwell JW, Kollman PA, Case DA (2004). Development and testing of a general amber force field.. J Comput Chem.

[32] Hornak V, Abel R, Okur A, Strockbine B, Roitberg A (2006). Comparison of multiple Amber force fields and development of improved protein backbone parameters.. Proteins.

[33] Phillips JC, Braun R, Wang W, Gumbart J, Tajkhorshid E (2005). Scalable molecular dynamics with NAMD.. Journal of computational chemistry.

[34] Christen M, Hunenberger PH, Bakowies D, Baron R, Burgi R (2005). The GROMOS software for biomolecular simulation: GROMOS05.. J Comput Chem.

[35] Daura X, Gademann K, Jaun B, Seebach D, van Gunsteren WF (1999). Peptide Folding: When Simulation Meets Experiment.. Angew Chem Int Ed.

[36] Duschak VG, Couto AS (2009). Cruzipain, the major cysteine protease of Trypanosoma cruzi: a sulfated glycoprotein antigen as relevant candidate for vaccine development and drug target. A review.. Curr Med Chem.

[37] Gehlhaar DK, Verkhivker GM, Rejto PA, Sherman CJ, Fogel DB (1995). Molecular recognition of the inhibitor AG-1343 by HIV-1 protease: conformationally flexible docking by evolutionary programming.. Chem Biol.

[38] Amaro RE, Baron R, McCammon JA (2008). An improved relaxed complex scheme for receptor flexibility in computer-aided drug design.. Journal of computer-aided molecular design.

[39] Lin JH, Perryman AL, Schames JR, McCammon JA (2002). Computational drug design accommodating receptor flexibility: the relaxed complex scheme.. Journal of the American Chemical Society.

[40] Amaro RE, Schnaufer A, Interthal H, Hol W, Stuart KD (2008). Discovery of drug-like inhibitors of an essential RNA-editing ligase in Trypanosoma brucei.. Proceedings of the National Academy of Sciences.

[41] Bairoch A, Apweiler R, Wu CH, Barker WC, Boeckmann B (2005). The Universal Protein Resource (UniProt).. Nucleic Acids Research.

[42] Rawlings ND, Morton FR, Kok CY, Kong J, Barrett AJ (2008). MEROPS: the peptidase database.. Nucleic Acids Res.

[43] Berman HM, Westbrook J, Feng Z, Gilliland G, Bhat TN (2000). The Protein Data Bank.. Nucleic Acids Research.

[44] Larkin MA, Blackshields G, Brown NP, Chenna R, McGettigan PA (2007). Clustal W and Clustal X version 2.0.. Bioinformatics.

[45] Roberts E, Eargle J, Wright D, Luthey-Schulten Z (2006). MultiSeq: unifying sequence and structure data for evolutionary analysis.. BMC bioinformatics.

[46] Humphrey W, Dalke A, Schulten K (1996). VMD: visual molecular dynamics.. J Mol Graph.

[47] Russell RB, Barton GJ (1992). Multiple protein sequence alignment from tertiary structure comparison: assignment of global and residue confidence levels.. Proteins.

[48] Brinen LS, Hansell E, Cheng J, Roush WR, McKerrow JH (2000). A target within the target: probing cruzain's P1′ site to define structural determinants for the Chagas' disease protease.. Structure.

[49] Turk D, Guncar G, Podobnik M, Turk B (1998). Revised definition of substrate binding sites of papain-like cysteine proteases.. Biol Chem.

[50] Douc-Rasy S, Riou JF, Ahomadegbe JC, Riou G (1988). ATP-independent DNA topoisomerase II as potential drug target in trypanosomes.. Biol Cell.

[51] Pate PG, Wolfson JS, McHugh GL, Pan SC, Swartz MN (1986). Novobiocin antagonism of amastigotes of Trypanosoma cruzi growing in cell-free medium.. Antimicrob Agents Chemother.

[52] Kerr ID, Lee JH, Farady CJ, Marion R, Rickert M (2009). Vinyl sulfones as antiparasitic agents and a structural basis for drug design.. J Biol Chem.

[53] Gillmor SA, Craik CS, Fletterick RJ (1997). Structural determinants of specificity in the cysteine protease cruzain.. Protein Sci.

[54] Allende LM, Garcia-Perez MA, Moreno A, Corell A, Carasol M (2001). Cathepsin C gene: First compound heterozygous patient with Papillon-Lefevre syndrome and a novel symptomless mutation.. Hum Mutat.

[55] Yang Y, Bai X, Liu H, Li L, Cao C (2007). Novel mutations of cathepsin C gene in two Chinese patients with Papillon-Lefevre syndrome.. J Dent Res.

[56] Hart TC, Hart PS, Michalec MD, Zhang Y, Firatli E (2000). Haim-Munk syndrome and Papillon-Lefevre syndrome are allelic mutations in cathepsin C.. J Med Genet.

[57] Toomes C, James J, Wood AJ, Wu CL, McCormick D (1999). Loss-of-function mutations in the cathepsin C gene result in periodontal disease and palmoplantar keratosis.. Nat Genet.

