# Supplementary material for: Computational Identification of Uncharacterized Cruzain Binding Sites
Source: PLoS Negl Trop Dis. 2010 May 11;4(5):e676. doi: 10.1371/journal.pntd.0000676 (PMC2867933; doi:10.1371/journal.pntd.0000676)
Supplement: Figure S1 — The alignment of selected peptidase C1 family members. (0.18 MB PDF) [file pntd.0000676.s001.pdf]

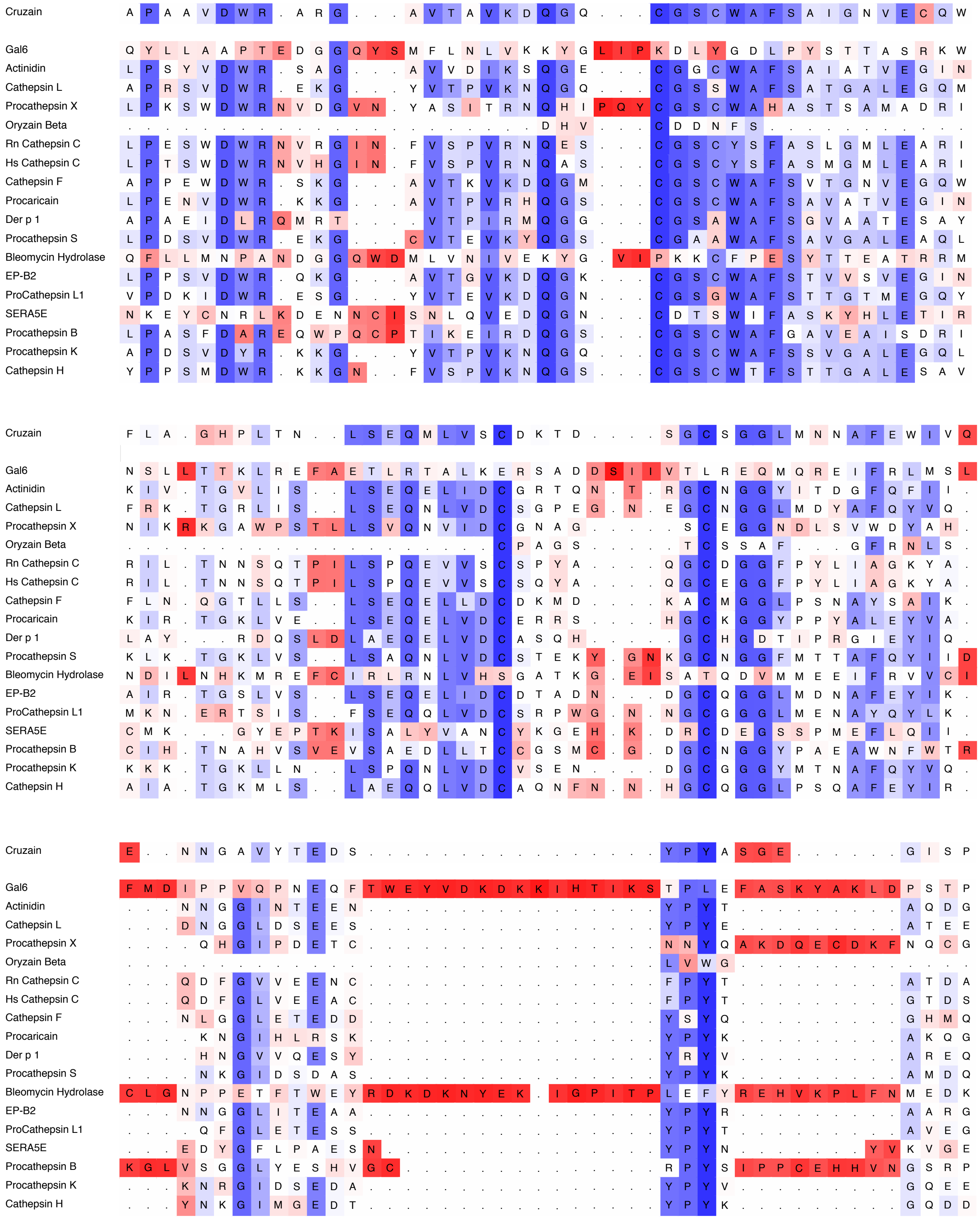
Cruzain APAAVDWR.ARG...AVTAVKDQGQ...CGSCWAFSAIGNVECQW
Gal6 QYLLAAPTEDGGQYSMFLNLVKKYGLIPKDLYGDLPYSTTASRKW
Actinidin LPSYVDWR.SAG...AVVDIKSQGE...CGGCWAFSAIATVEGIN
Cathepsin L APRSVDWR.EKG...YVTPVKNQGQ...CGSSWAFSATGALEGQM
Procathepsin X LPKSWDWRNVDGVN.YASITRNQHIPQYCGSCWAHASTSAMADRI
Oryzain Beta ......................DHV...CDDNFS...........
Rn Cathepsin C LPESWDWRNVRGIN.FVSPVRNQES...CGSCYSFASLGMLEARI
Hs Cathepsin C LPTSWDWRNVHGIN.FVSPVRNQAS...CGSCYSFASMGMLEARI
Cathepsin F APPEWDWR.SKG...AVTKVKDQGM...CGSCWAFSVTGNVEGQW
Procaricain LPENVDWR.KKG...AVTPVRHQGS...CGSCWAFSAVATVEGIN
Der p 1 APAEIDLRQMRT....VTPIRMQGG...CGSAWAFSGVAATESAY
Procathepsin S LPDSVDWR.EKG...CVTEVKYQGS...CGAAWAFSAVGALEAQL
Bleomycin Hydrolase QFLLMNPANDGGQWDMLVNIVEKYG.VIPKKCFPESYTTEATRRM
EP-B2 LPPSVDWR.QKG...AVTGVKDQGK...CGSCWAFSTVVSVEGIN
ProCathepsin L1 VPDKIDWR.ESG...YVTEVKDQGN...CGSGWAFSTTGTMEGQY
SERA5E NKEYCNRLKDENNCISNLQVEDQGN...CDTSWIFASKYHLETIR
Procathepsin B LPASFDAREQWPQCPTIKEIRDQGS...CGSCWAFGAVEAISDRI
Procathepsin K APDSVDYR.KKG...YVTPVKNQGQ...CGSCWAFSSVGALEGQL
Cathepsin H YPPSMDWR.KKGN..FVSPVKNQGS...CGSCWTFSTTGALESAV

Cruzain FLA.GHPLTN..LSEQMLVSCDKTD....SGCSGGLMNNAFEWIVQ
Gal6 NSLLTTKLREFAETLRTALKERSADDSIIVTLREQMQREIFRLMSL
Actinidin KIV.TGVLIS..LSEQELIDCGRTQN.T.RGCNGGYITDGFQFII.
Cathepsin L FRK.TGRLIS..LSEQNLVDCSGPEG.N.EGCNGGLMDYAFQYVQ.
Procathepsin X NIKRKGAWPSTLLSVQNVIDCGNAG......SCEGGNDLSVWDYAH.
Oryzain Beta ....................CPAGS.....TCSSAF...GFRNLS.
Rn Cathepsin C RIL.TNNSQTPILSPQEVVSCSPYA....QGCDGGFPYLIAGKYA.
Hs Cathepsin C RIL.TNNSQTPILSPQEVVSCSQYA....QGCEGGFPYLIAGKYA.
Cathepsin F FLN.QGTLLS..LSEQELLDCDKMD....KACMGGLPSNAYSAIK.
Procaricain KIR.TGKLVE..LSEQELVDCERRS....HGCKGGYPPYALEYVA.
Der p 1 LAY...RDQSLDLAEQELVDCASQH.....GCHGDTIPRGIEYIQ.
Procathepsin S KLK.TGKLVS..LSAQNLVDCSTEKY.GNKGCNGGFMTTAFQYIID
Bleomycin Hydrolase NDILNHKMREFCIRLRNLVHSGATKG.EISATQDVMMEEIFRVVCI
EP-B2 AIR.TGSLVS..LSEQELIDCDTADN...DGCQGGLMDNAFEYIK.
ProCathepsin L1 MKN.ERTSIS..FSEQQLVDCSRPWG.N.NGCGGGLMENAYQYLK.
SERA5E CMK...GYEPTKISALYVANCYKGEH.K.DRCDEGSSPMEFLQII.
Procathepsin B CIH.TNAHVSVEVSAEDLLTCCGSMC.G.DGCNGGYPAEAWNFWTR
Procathepsin K KKK.TGKLLN..LSPQNLVDCVSEN....DGCGGGYMTNAFQYVQ.
Cathepsin H AIA.TGKMLS..LAEQQLVDCAQNFN.N.HGCQGGLPSQAFEYIR.

Cruzain E..NNGAVYTEDS................YPYASGE......GISP
Gal6 FMDIPPVQPNEQFTWEYVDKDKKIHTIKSTPLEFASKYAKLDPSTP
Actinidin ...NNGGINTEEN................YPYT.........AQDG
Cathepsin L ...DNGGLDSEES................YPYE.........ATEE
Procathepsin X ....QHGIPDETC................NNYQAKDQECDKFNQCG
Oryzain Beta ....LVWG..............
Rn Cathepsin C ...QDFGVVEENC................FPYT.........ATDA
Hs Cathepsin C ...QDFGLVEEAC................FPYT.........GTDS
Cathepsin F ...NLGGLETEDD................YSYQ.........GHMQ
Procaricain ....KNGIHLRSK................YPYK.........AKQG
Der p 1 ....HNGVVQESY................YRYV.........AREQ
Procathepsin S ....NKGIDSDAS................YPYK.........AMDQ
Bleomycin Hydrolase CLGNPPETFTWEYRDKDKNYEK.IGPITPLEFYREHVKPLFNMEDK
EP-B2 ...NNGGLITEAA................YPYR.........AARG
ProCathepsin L1 ....QFGLETESS................YPYT.........AVEG
SERA5E ...EDYGFLPAESN...............YPYN.......YVKVGE
Procathepsin B KGLVSGGLYESHVGC..............RPYSIPPCEHHVNGSRP
Procathepsin K ...KNRGIDSEDA................YPYV.........GQEE
Cathepsin H ...YNKGIMGEDT................YPYK.........GQDD

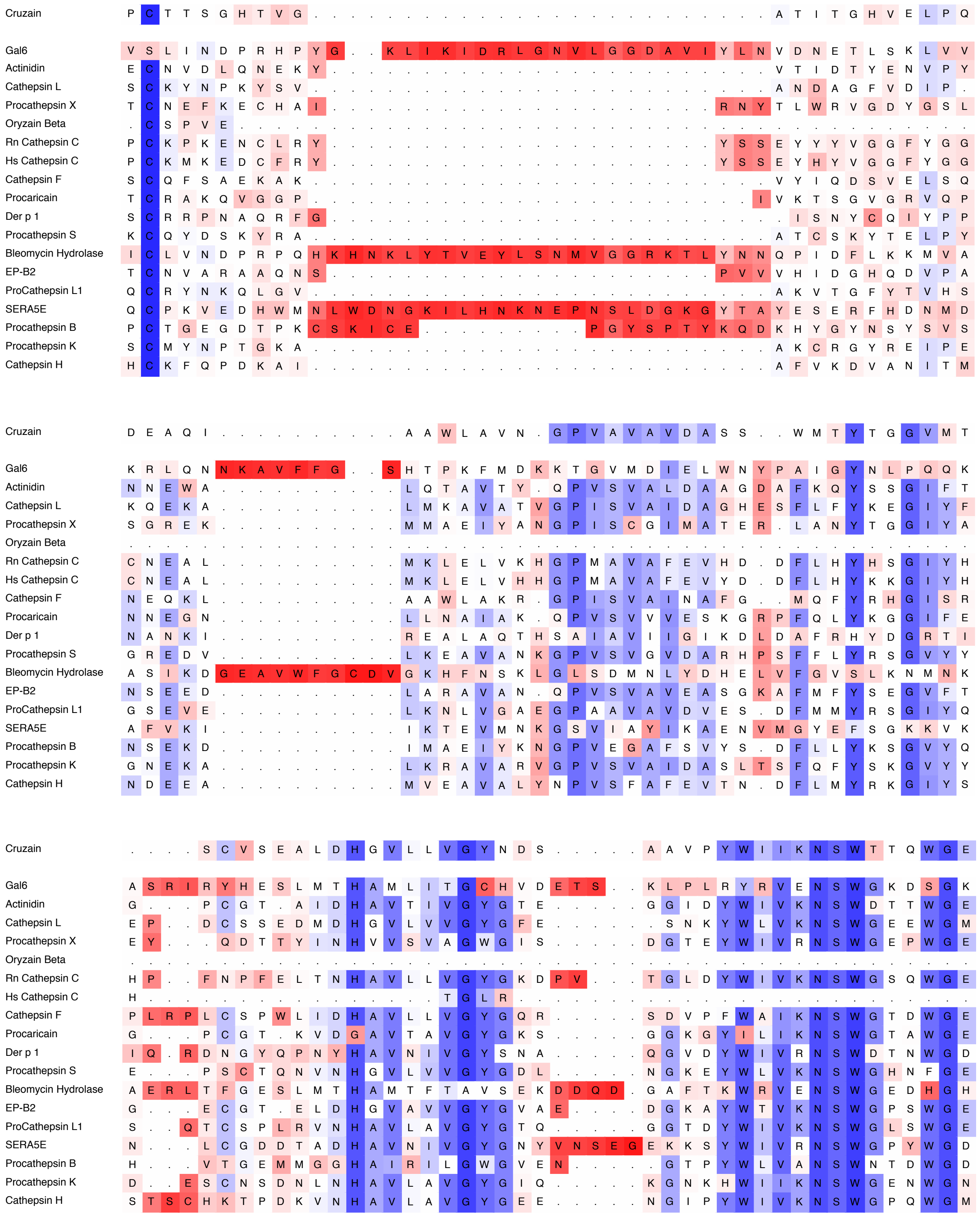
Cruzain
Gal6
Actinidin
Cathepsin L
Procathepsin X
Oryzain Beta
Rn Cathepsin C
Hs Cathepsin C
Cathepsin F
Procaricain
Der p 1
Procathepsin S
Bleomycin Hydrolase
EP-B2
ProCathepsin L1
SERA5E
Procathepsin B
Procathepsin K
Cathepsin H

Cruzain	E	G	Y	I	R	I	A	K	.	.	.	.	.	.	G	S	N	Q	C	L	V	K	E	E	A	S	S	A	V	V	G
Gal6	D	G	L	Y	V	M	T	Q	K	Y	F	E	E	Y	C	F	Q	I	V	V	D	I	N	E	L	P	K	E	L	A	S
Actinidin	E	G	Y	M	R	I	L	R	N	V	.	.	.	G	G	A	G	T	C	G	I	A	T	M	P	S	Y	P	V	K	Y
Cathepsin L	G	G	Y	V	K	M	A	K	D	.	.	.	.	.	R	R	N	H	C	G	I	A	S	A	A	S	Y	P	T	V	.
Procathepsin X	R	G	W	L	R	I	V	T	S	T	Y	K	D	G	K	G	A	R	Y	N	L	A	I	E	E	H	C	T	F	G	D
Oryzain Beta	.	.	.	.	.	.	.	.	.	.	.	.	.	.	.	.	.	.	.	.	.	.	.	.	.	.	.	.	.	.	.
Rn Cathepsin C	S	G	Y	F	R	I	R	R	G	.	.	.	.	.	.	T	D	E	C	A	I	E	S	I	A	M	A	A	I	P	I
Hs Cathepsin C	.	.	.	.	.	.	.	.	.	.	.	.	.	.	.	.	.	.	.	.	.	.	.	.	.	.	.	.	.	.	.
Cathepsin F	K	G	Y	Y	Y	L	H	R	.	.	.	.	.	.	G	S	G	A	C	G	V	N	T	M	A	S	S	A	V	V	D
Procaricain	K	G	Y	I	R	I	K	R	A	P	G	.	.	N	S	P	G	V	C	G	L	Y	K	S	S	Y	Y	P	T	K	N
Der p 1	N	G	Y	G	Y	F	A	A	N	.	.	.	.	.	.	I	D	L	M	M	I	E	E	Y	P	Y	V	V	I	L	G
Procathepsin S	E	G	Y	I	R	M	A	R	N	.	.	.	.	.	K	G	N	H	C	G	I	A	S	F	P	S	Y	P	E	I	.
Bleomycin Hydrolase	K	G	Y	L	C	M	T	D	E	W	F	S	E	Y	V	Y	E	V	V	V	D	R	K	H	V	P	E	E	V	L	A
EP-B2	Q	G	Y	I	R	V	E	K	D	S	G	.	.	A	S	G	G	L	C	G	I	A	M	E	A	S	Y	P	V	K	T
ProCathepsin L1	R	G	Y	I	R	M	V	R	N	.	.	.	.	.	R	G	N	M	C	G	I	A	S	L	A	S	L	P	M	V	A
SERA5E	E	G	Y	F	K	V	D	M	Y	G	.	.	.	.	.	P	T	H	C	H	F	N	F	I	H	S	V	V	I	F	N
Procathepsin B	N	G	F	F	K	I	L	R	G	.	.	.	.	.	.	Q	D	H	C	G	I	E	S	E	V	V	A	G	I	P	R
Procathepsin K	K	G	Y	I	L	M	A	R	N	.	.	.	.	.	K	N	N	A	C	G	I	A	N	L	A	S	F	P	K	M	.
Cathepsin H	N	G	Y	F	L	I	E	R	.	.	.	.	.	.	G	K	N	M	C	G	L	A	A	C	A	S	Y	P	I	P	L
